# Leptin Functions in Infectious Diseases

**DOI:** 10.3389/fimmu.2018.02741

**Published:** 2018-11-26

**Authors:** Radheshyam Maurya, Parna Bhattacharya, Ranadhir Dey, Hira L. Nakhasi

**Affiliations:** ^1^Department of Animal Biology, School of Life Science, University of Hyderabad, Hyderabad, India; ^2^Division of Emerging and Transfusion Transmitted Diseases, Center for Biologics Evaluation and Research, Food and Drug Administration, Silver Spring, MD, United States

**Keywords:** leptin, leishmaniasis, trypanosomiasis, amoebiasis, malaria, bacteria, virus, malnutrition

## Abstract

Leptin, a pleiotropic protein has long been recognized to play an important role in the regulation of energy homeostasis, metabolism, neuroendocrine function, and other physiological functions through its effects on the central nervous system (CNS) and peripheral tissues. Leptin is secreted by adipose tissue and encoded by the obese (*ob*) gene. Leptin acts as a central mediator which regulates immunity as well as nutrition. Importantly, leptin can modulate both innate and adaptive immune responses. Leptin deficiency/resistance is associated with dysregulation of cytokine production, increased susceptibility toward infectious diseases, autoimmune disorders, malnutrition and inflammatory responses. Malnutrition induces a state of immunodeficiency and an inclination to death from communicable diseases. Infectious diseases are the disease of poor who invariably suffer from malnutrition that could result from reduced serum leptin levels. Thus, leptin has been placed at the center of many interrelated functions in various pathogenic conditions, such as bacterial, viruses and parasitic infections. We review herein, the recent advances on the role of leptin in malnutrition in pathogenesis of infectious diseases with a particular emphasis on parasitic diseases such as Leishmaniasis, Trypanosomiasis, Amoebiasis, and Malaria.

## Introduction

Leptin is a hormone derived from adipocytes in response to the nutritional status, and it signals to the central nervous system (CNS) and peripheral organs (1). The circulating plasma leptin concentrations are mostly influenced by the total body fat mass index, metabolic hormones, and gender. Women have higher concentrations of circulating leptin compared to men (2). The central function of leptin is metabolic homeostasis that can be attained by the delivery of information about the total body fat mass to the hypothalamus that in turn alters the CNS function and regulates glucocorticoids, insulin hormone and food intake & energy balance (3, 4). Concurrently, leptin is also a critical regulator of immunity and functions as a pro-inflammatory cytokine-like interleukin (IL)-1, IL-6, IL-8, IL-18, and tumor necrosis factor-α (TNF-α), and its deficiency increases susceptibility to infectious (5–8).

Leptin was identified as the gene defect responsible for the obesity syndrome in Leptin-deficient (*ob/ob*) mice and reported as the product of *Ob* gene (9, 10). Leptin is a 16 kDa α-helix type protein like the long-chain helical cytokine family such as IL-6, IL-2, IL-12, leukocyte inhibitory factor (LIF), Granulocyte-colony stimulating factor (G-CSF), Ciliary neurotrophic factor (CNTF), and Oncostatin M (11, 12). Most of the biological functions of leptin are exerted through leptin receptor (Ob-R) signaling via the Janus kinase/signal transducer and activator of transcription (JAK/STAT) pathway (13, 14). In general, leptin enhances the immune response via activating antigen presenting cells (APCs), Th1 cells function and proliferation, and mediating the secretion of the pro-inflammatory cytokines, such as TNF-α, IL-2, or IL-6 (7, 15, 16). Leptin-deficient (*ob/ob*; double knockout obese gene) and leptin receptor-deficient (*db/db*; double knockout obese receptor gene) mice display marked reduction in the size & thymic atrophy and exhibit defective immune responses (17, 18). Similarly, reduced leptin levels in starved and malnourished individuals is further associated with alterations of the immune response and thymic atrophy. However, these conditions can be reversed by leptin administration (19–22).

Over the past decade, the role leptin in infectious diseases was extensively explored. It has been reported that leptin deficiency is correlated with starvation or nutritional deprivation/malnutrition (23). Malnutrition affects both innate & acquired immunity of the host (24) thereby increasing the incidences of infections and mortality (25). The immune dysfunction in malnutrition or restricted calorie intake reduced the memory T cells, total CD4+ and CD8+ T cell numbers compared to well-nourished infected controls (26–29). Interestingly, leptin has a crucial role in mediating phagocytosis, T cell number, function, and metabolism in both obesity and malnutrition. The systemic circulating leptin deficiency in malnutrition is also correlated with several other bacterial, viral and parasitic infections such as tuberculosis (30), pneumonia (31), sepsis (32), colitis (33), viral infection (34, 35) leishmaniasis (36), trypanosomiasis (37), amoebiasis (38), and malaria (39) due to defective cytokine production (40–45). Hence, nutritional status is critically essential for immune cell function in both malnutrition and infection. Understanding how leptin is altered in malnutrition and infection will lead to better insight of and treatment for diseases where nutritional status determines clinical outcome. Furthermore, there has been increasing evidence that leptin is involved in the pathogenesis of various infectious diseases. In the present review, we will discuss the emerging role of leptin in different infectious diseases and will further highlight how malnutrition or starvation could play a role.

## Leptin and immunity

Leptin is a pleiotropic molecule, which can function as a hormone as well as cytokine (adipokine). Almost all immune cells such as neutrophils, monocytes, lymphocytes express leptin receptor and it belongs to the family of class-I cytokine receptors (46–48). Leptin regulates angiogenesis, hematopoiesis, innate & adaptive immunity and induces the Th1 response by increasing IFN-γ, IL-2, and TNF-α production, subsequently leading to the activation of monocyte/macrophages and prevents the apoptosis of various immune cells by delaying the cleavage of Bid and Bax (49–55).

In innate immunity, leptin enhances the activity and function of neutrophils by the release of oxygen free radicals, increased CD11b expression and intercellular adhesion molecule-1 (ICAM-1), which leads to migration of immune cells at the sites of inflammation (56–58). Leptin activates the monocytes and dendrite cells (DCs) that in turn leads to the production of pro-inflammatory cytokines such as TNF-α, IL-6 along with IL-12, a key cytokine that facilitates the shifting of T-cells toward the Th1 phenotype (59–62). Leptin also promotes DCs survival by triggering the activation of nuclear factor-kappa B (NF-kappa B) and up-regulates B-cell lymphoma 2 (Bcl-2) and B-cell lymphoma-extra-large (Bcl-xL) gene expression via the PI3K-Akt signaling pathway (62). Moreover, upon leptin stimulation, DCs also exhibit increased production of multiple cytokines including IL-1, IL-6, IL-12, TNF-α, MIP-1α and induces the expression of surface molecules, such as CD1a, CD80, CD83, or CD86 (63, 64). Indirectly, leptin leads to the activation of natural killer (NK) cells upon modulation of IL-1, IL-6, and TNF-α via monocytes and macrophages (61) resulting an increased IL-12 and reduced IL-15 expression in NK cells (65, 66).

**Figure 1:**
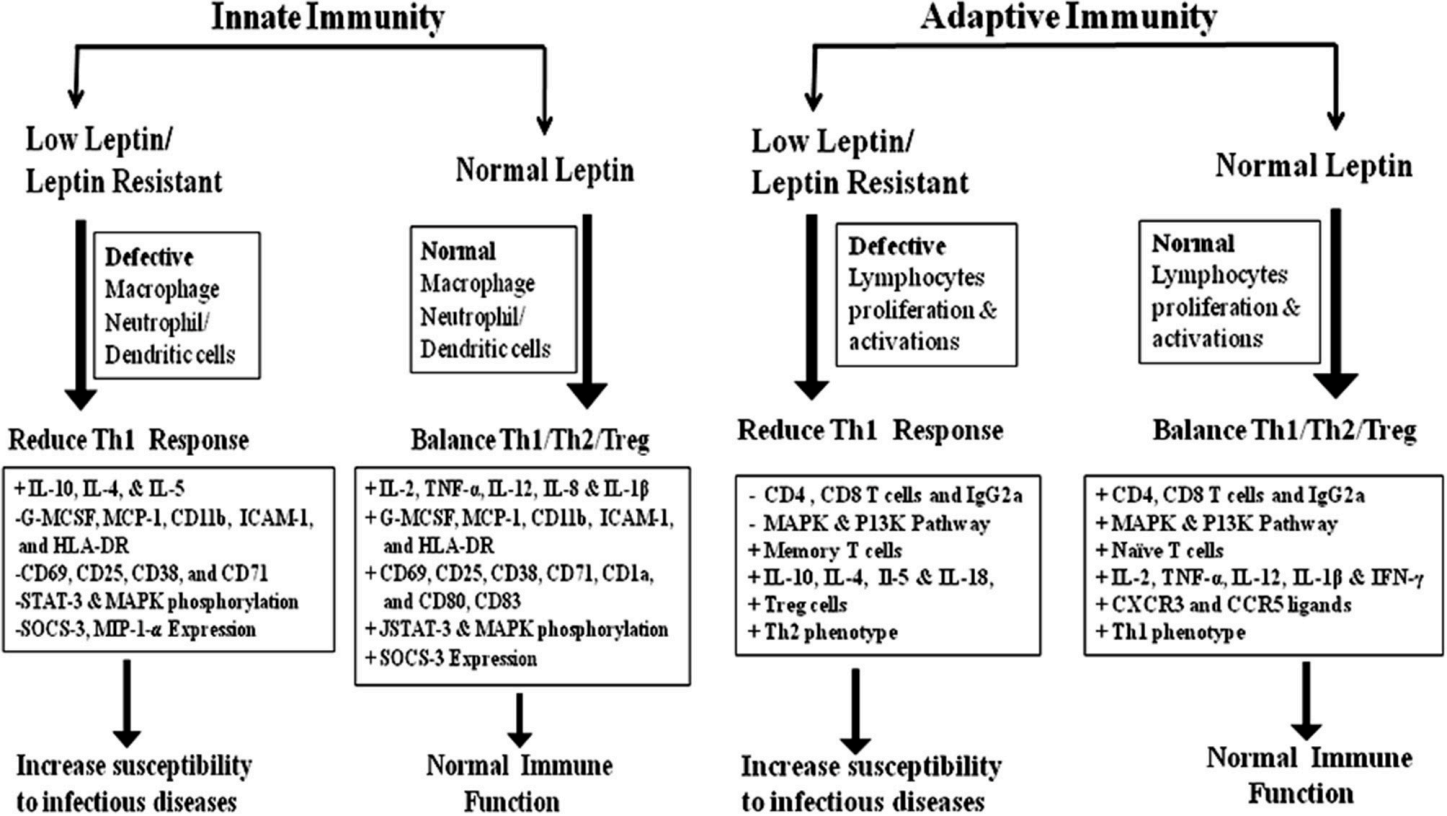
The role of leptin in Innate and adaptive immunity: innate immunity; Leptin plays a crucial role in the activation and proliferation of macrophage, neutrophil, and dendritic cells through the up and down regulation of various cytokine/chemokines. Adaptive immunity; Leptin-induced the activation and proliferation of total lymphocytes (T and B cells), regulatory T cells and Naive T cells through the up and down regulation of pro-inflammatory and anti-inflammatory cytokines. The Innate and adaptive immune response induced by leptin signaling through the phosphorylation of MAKP/STAT-3/P13K pathways (15–18, 49–55).

In adaptive immunity, leptin induces the maturation and survival of thymic T-cells by reducing their rate of apoptosis through inhibition of FAS-directed apoptosis pathway (16, 67). Eventually, leptin has anti-apoptotic effects on mature T-cells, by up-regulating the expression of Bcl-xL (68), T-box transcription factor (T-bet) (69), and synergizes with other cytokines in lymphocyte proliferation and activation possibly via signal transducer and activator of transcription 3 (STAT3) signaling (70, 71). Leptin deficiency in both mouse and human results in severe immune defects characterized by decrease in total lymphocytes, CD4+ helper T cell number, increased thymocyte apoptosis, and a skewing away from the Th1 toward Th2 phenotype thereby resulting in increased susceptibility to intracellular infections (16, 17, 72–74). Leptin mediates T-cells polarization by inducing the cell-mediated immune response through the secretion of IL-2, IL-12, TNF-α, and IFN-γ from Th1 cells and suppresses the production of IL-10 and IL-4 from Th2 cells (13, 75–78). In facts, thymocytes treated with leptin induces CD4+CD8+ cell differentiation mainly to CD4+ mature thymocytes (79). Additionally, leptin also activates human B cells to secrete cytokines, such as IL-6, IL-10, and TNF-α, through the activation of JAK2/STAT3 and p38MAPK/ERK1/2 signaling pathways (80, 81). Leptin-STAT3 signaling also influences the production of C-X-C chemokine receptor type 3 (CXCR3) and C-C chemokine receptor type 5 (CCR5) ligands, which are preferentially expressed on Th1 cells and enhances pro-inflammatory cytokines such as IL-1β, TNF-α, and IL-6 in serum (39, 82, 83). In conclusion, leptin acts as a Th1 cytokine and regulates all immune cells through leptin receptor and affects innate & adaptive immune responses together facilitating a shift toward Th1 response. The brief role of leptin in innate & adaptive immune response was summarized in Figure 1.

## Leptin in malnutrition

Nutritional deficiency impaired phagocyte function, cell-mediated immunity, cytokine production, antibody response and the complement system (24, 25, 84) and predisposed to death from infectious diseases (85). Concurrently, malnutrition is the most common cause of secondary immunodeficiency worldwide associated with protein-energy malnutrition (PEM) (86, 87), a nutritional deficiency in which individuals suffer from protein but not calorific malnutrition (88). Strikingly, PEM causes a drastic reduction in body fat mass and decreases the circulating concentration of leptin, which, in turn, impairs the generation of proinflammatory mediators [IFN-γ, TNF-α and (NO) nitric oxide] (89, 90), and increases the incidence of infectious diseases (72, 83). Malnutrition is a primary risk factor for many infectious diseases. From recent research, it seems that malnutrition is a predictor of tuberculosis disease and is associated with worse outcomes. Active tuberculosis is correlated with weight loss, cachexia, and low serum concentrations of leptin which in turn suppresses the lymphocyte stimulation and Th1 cytokines such as IL-2, IFN-γ, and TNF-α secretion (91–96). PEM significantly reduced the lymphocyte stimulation as well as secretion of the Th1 cytokines such as IL-2, IFN-γ, and TNF-α, in M. tuberculosis-infected guinea pig (92, 97). Furthermore, PEM also diminishes leptin concentrations and increases serum levels of stress hormones, i.e., glucocorticoids which impairs macrophage functions by limiting NF-kB translocation into the nucleus (96). Macrophages from experimental PEM mice are less sensitive to lipopolysaccharides (LPS) due to decreased NF-kB translocation resulting in impairment of active phagocytosis, cytokines response and reactive oxygen intermediates (ROIs) productions (98–101).

Serum leptin concentration decreases as malnutrition becomes more pronounced and thus serves as a biomarker of poor nutritional status in chronic cirrhosis due to viral hepatitis and candidiasis due to *Candida albicans* (102–105). Moreover, during fasting or starvation, leptin levels also fall disproportionately due to the decrease in adipose tissue fat mass (106, 107). The decrease in leptin level during starvation rendered wild-type mice susceptible to LPS and TNF-α induced lethality whereas leptin treatment restores those changes despite ongoing starvation, suggesting that the lack of leptin plays a role in the immune dysfunction during starvation (18, 108). Exogenous leptin administration modulates T cell responses in mice and prevents starvation-induced immune suppression on the development of a delay type of hypersensitivity (DTH) response and protects from starvation-induced lymphoid atrophy in mice (12, 15, 68, 109).

Taken together, Leptin is a protein hormone secreted by adipocytes, regulating body fat and food intake through neuroendocrine-signaling system. Hence, it is possible to speculate that leptin might act in the brain to directly regulate metabolic response along with peripheral immune function thereby contributing to better outcomes in various infectious diseases compared with states of relative or total leptin deficiency. Importantly, recent studies reported that serum leptin level may be used as a promising diagnostic or prognostic marker for critical illness sepsis that is triggered by an infective agent such as bacteria, viruses, fungi, or parasites (110). Therefore, from here onwards, the review focuses on the role of leptin in various infectious diseases.

## Leptin and bacterial infections

Leptin-deficient mice and mice rendered leptin-deficient by fasting exhibit impaired pulmonary bacterial clearance (Table 1) and enhanced lethality during pulmonary tuberculosis, bacterial pneumonia, and sepsis (30–32). The patients with pulmonary tuberculosis (PTB) have decreased serum leptin levels, and an increase in adiponectin may serve as a reliable biomarker for predicting the development and progression of PTB pathogenesis (111, 145, 146). Mycobacterium infection in *ob/ob* mice was hampered to produce organized granulomatous response and defective in CD4+ and CD8+ T cells functions, IFN-γ and DTH responses (30). The mechanisms underlying the defective leukocyte effector function in cells from leptin-deficient mice were associated with a reduction in leukotriene (LT) synthesis in alveolar macrophage (AMs), reduced complement receptor (CR3) expression and decreased H_2_O_2_ synthesis in neutrophils (PMNs) infected with *Klebsiella pneumoniae* (112–114). Restoring the level of circulating leptin to physiological levels in fasted and *ob/ob* mice significantly improved the survival and pulmonary bacterial clearance, reduced bacteraemia, reconstituted alveolar macrophage phagocytosis and increased H_2_O_2_ production in the PMNs resulting in increased killing of *S. pneumonia in vitro* (31, 115). Leptin binding to leptin receptor activates multiple intracellular signaling pathways, including STAT3, STAT5, and ERK1/2. STAT3 activates transcription of suppressor of cytokine signaling (SOCS)-3, a protein that inhibits JAK2 and STAT3 signaling during prolonged stimulation of the Leptin receptor long isoform (*Lep-Rb*) (147, 148). *Lep-Rb* mediated phosphorylation of Tyr^1077^ activates STAT5 signaling pathway (149). Phosphorylation of Tyr^985^ in leptin receptor recruits binding partners SH2-containing tyrosine phosphatase (SHP-2) and growth factor binding 2 (GRB2) which activate extracellular signal-regulated kinase 1 and 2 (ERK 1/2) signaling (147, 150). A mutation of the Try^985^ with L^985^ residue in the leptin receptor exhibited increased mortality and impaired pulmonary bacterial clearance following an intratracheal challenge with *K. pneumoniae* due to the disruption of ERK-dependent activation (151).

**Table 1:** The distinctive immune responses in various infectious diseases upon leptin treatment were summarized.

**Infectious group**	**Infectious species**	**Effect of leptin on the Immune response**	**Model used (*in-vitro & in-vivo*)**	**References**
Bacterial disease	*Mycobacterium tuberculosis*	Increase IFNγ & TNF-α levels and PMN cells & functions. Increase T helper CD4 T cells & CD8 T cells activity. Improve Ag- specific antibody response. Restored DTH response and Granuloma formation. Reduced IL-6 cytokine and bacterial load.	Mice and human	(30, 95, 111)
	*Klebsiella pneumonia*	Increase phagocytosis index and Leukotriene synthesis. Improve defective alveolar macrophage phagocytosis. Restored CD11b expression level. Decrease bacterial load and reduce mortality.	Mice	(112, 113)
	*Pneumococcal pneumonia*	Restored defective alveolar macrophage phagocytosis activity. Increase PMN H_2_O_2_ production. Reduced TNF-α, MIP-2, PGE2 in the lung. Improves pulmonary bacterial clearance and survival.	Mice	(31, 114)
	*Clostridium difficile*	Leptin receptor Q223R mutation leads to defective STAT3 signaling pathway and associated with an increased risk of colitis. Mutation of tyrosine 1,138 in the intracellular domain of LepRb decreased mucosal chemokine and cell recruitment. Increases inflammation, colonic chemokine expression, and cellular recruitment. Improve the bacterial clearance.	Mice	(33)
	Sepsis	Improve the Neutrophil function. Increase the phosphorylation of p38 MAP kinase. Control sepsis-induced organ damage Supresses IL-6 and MCP-1 level. Control the bacteraemia.	Mice and rat	(32, 110, 115)
	*Listeria monocytogenes*	Induce CD11b expression on neutrophils and lower the apoptosis. Induce effective bacterial phagocytosis and lymphocytic apoptosis in sever immune-deficiency. Improvement of anti-listerial resistance and the MCP-1 mRNA expression. Decrease defective MCP-1 expression in the liver. Control the bacteraemia.	Mice	(116, 117)
	*Helicobactor pylori*	Increase mucosal leptin in the infected patients compare to uninfected patients. Amount of gastric leptin correlated positively with the mucosal levels of IL-1β and IL-6, but not IL-8 cytokine. Increase of gastric leptin expression during infection may have a local rather than systemic action. Increase in serum leptin concentration. Circulating leptin correlated with body mass index, but not with bacterial load. There was no change in plasma leptin levels following cure of the infection.	Mice and human	(118–121)
Viral disease	Influenza A/H1N1 pneumonia	Global deficiency of leptin receptor (db/db) have worsened survival following influenza A infection. Leptin receptor deficiency impaired viral clearance & diminished the IFN-γ levels. Loss of leptin receptor within lung epithelium or within macrophages is not associated with worsened lung injury or mortality following infection. Decrease proinflammatory cytokines IL-6 and IL-1β level and increase survival. Disruption of leptin signaling in T cells limits worsened the pH1N1 dependent mortality and infection severity.	Human and mice	(34, 122–125)
	Respiratory Syncytial Virus	Promoted Th17 subset differentiation. Suppressed Th2 subset differentiation. Increased phosphorylation of ERK1/2 in peripheral Lymphocytes.	Human	(126)
	HIV	Leptin inhibits ROS and control oxidative burst mechanism in HIV+ monocyte patients. Leptin receptor (ob-R) expression increased in HIV+ PBMCs than control. Serum leptin level positively correlated with CD4+ T lymphocyte during antiviral therapy in HIV patients. Supresses SOCS3 & mTOR expression and Th2 subset differentiation. Reduced viral load.	Human and mice (*in-vitro* & *in-vivo*)	(35, 127–129)
Parasitic disease	*Leishmania major/Leishmania donovani*	Activates macrophage phagocytosis and ROS induction. Enhances the phosphorylation of Erk1/2 & Akt in macrophages. Increases IFN-γ, IL12, IL-1β secretion in macrophage. Improve IFN-γ/IL-10 ratio, GrzA and Th1 cytokine response. Activate CD8+ T-cell compartment and reduces PD-1 & CTLA-4 expression. Increase IgG2a levels and improve IgG2a/IgG1 ratio. Improve granuloma formation and repaired tissue degeneration. Reduced parasite load in visceral organs.	Human (THP-1 and PBMCs) Mice (*in-vitro* & *in-vivo*)	(36, 49, 130–132)
	*Trypanosoma cruzi*	Defective leptin receptors or reduction in leptin level increase parasitemia and mortality rate. Reconstitution of central leptin signaling in brain reduces tissue parasitism and mortality rates. Improve plasma cytokines and chemokine's.	Mice	(37, 133–136)
	*Entamoeba histolytica*	Mutation in leptin receptor (LEPR Q223R). Substitution of arginine (223R) in the cytokine receptor homology domain 1 of LEPR are more susceptible than those have glutamine (223Q) amino acid. Q223R polymorphism also decreased leptin-dependent STAT3 activation and defective STAT3 signaling and increase susceptibility to liver & intestinal abscess. Q223R leptin receptor mutation results in defective neutrophil infiltration to the site of infection. Mutation of tyrosine 985 or 1138 in leptin receptor results in defective SHP2/ERK and STAT3 signaling. Leptin-mediated resistance to amebiasis requires leptin receptor signaling through both the STAT3 and SHP2/ERK pathways. Leptin promotes regeneration & mucin secretion by epithelial cell and control apoptosis & integrity in intestinal epithelium lining. Low serum leptin increase liver and intestinal abscess. Intestinal parasites deregulate the secretion of leptin and adiponectin and play a role in enteric parasitosis by modulating body immunity, food intake and blood chemistry.	Human and mice	(38, 137–142)
	*Plasmodium berghei ANKA parasite*	Higher serum leptin levels. Increase mTORC1 (Mechanistic target of rapamycin complex 1) activity in CD4+ and CD8+ T cells in a dose dependent manner. Leptin act as downstream target for mTORC1 activity in T cells during ECM. The *leptin* gene mutation in *ob/ob* is associated with observed CM resistance phenotype. CM resistance phenotype is due to involvement of Th1 cytokines TNF-α and INF-γ in the regulatory cascade controlling inflammatory responses after malarial infections.	Mice	(39, 143, 144)

Moreover, Leptin-dependent neutrophilic phagocytosis of *L. monocytogenes* was more potent than *Escherichia coli* due to the presence of apoptotic factor Listeriolysin O, which is absent in *E. coli* (116). Exogenous leptin restored the anti-listeria resistance and monocyte chemoattractant protein-1 (MCP-1) and MIP-2 production in leptin-deficient mice (117, 152). *Clostridium difficile colitis* is a primary causative agent of nosocomial infection in humans and murine. The defective STAT3 signaling pathway leads to susceptibility to infectious colitis and bacterial peritonitis (33, 153) and leptin treatment restored the protective mucosal immune response in *C. difficile colitis* by the STAT3 inflammatory pathway (33).

In contrast to above finding, disruption of leptin receptor-mediated STAT3 signaling pathway improved AMs phagocytosis and host defense against *P. pneumonia* in (*Lepr*^*s1138/s1138*^) s/s mice following an intratracheal challenge with *S. pneumonia* (154). These effects are mediated by an intracellular signaling pathway that is dependent on ERK1/2 activation in AMs resulting an increased in LT synthesis, which enhanced the phagocytosis in cells from *s/s* mice (154). Mice infected with *Helicobacter pylori*-induced pro-inflammatory cytokine response and enhanced the leptin secretion from gastric mucosa which may be playing a role in weight gain after eradication of *H pylori* infection (118–120) suggesting that leptin has a local effect rather than systemic action in patients with gastritis (121, 155). These findings reveal the existence of a relevant neuroendocrine control of leptin in systemic immune defense in various bacterial diseases thereby highlighting the possible therapeutic potential of leptin analogous to control infectious diseases.

## Leptin and viral infections

Mice deficient in the leptin receptor or malnourished leads to impaired viral clearance (Table 1), diminished lung IFNγ level and reduced survival during influenza-A pneumonia infections (156). The mice lacking functional leptin receptor in T cells (LepR^T−/−^) limits pH1N1 influenza mortality and infection severity in obese mice suggesting that leptin signaling in T cells may be a critical mediator of pH1N1 severity in obese mice (122, 123). Moreover, Leptin resistant obese mice or decreased leptin level in obese individuals may increase the susceptibility to influenza virus infection by suppressing the memory T-cell function and IFN-α, IFN-β, and IFN-γ mRNA expression which leads to an increase in viral titer and infiltration (124). Furthermore, mice lacking functional leptin receptor in tissue- specific lung epithelial and macrophage cells have improved viral clearance and reduced lung injury following influenza-A infection suggesting that leptin signaling is also associated with non-myeloid cells such as natural killer cells and T cells (125). Leptin significantly upregulated the Th17 subset but suppressed Th2 subset differentiation possibly via regulating ERK1/2 phosphorylation in human bronchial epithelial cells (hBECs) infected with the respiratory syncytial virus (RSV) (126). Human immunodeficiency virus (HIV)-infected patients have an exaggerated expression of leptin receptor on their blood mononuclear cells while low leptin levels in their serum leads to an immune deficiency in these patients (127, 128). Importantly, leptin therapy, a novel strategy now in clinical trials, and its beneficial positive role in HIV patients in correction of metabolic complications related to HIV-associated lipodystrophy syndrome (HALS) has been reported recently (129). Leptin also diminishes the oxidative status of monocytes suggesting that leptin can alter the redox status of monocytes, which leads to immunological alterations in HIV infection (35). Therefore, taken together these findings reveal that leptin could control the systemic immune defense failure in viral specific immune cells dysfunction and further suggests the possible healing potential for leptin analogs in infectious disease.

## Leptin and parasitic infections

Parasitic infections contribute significantly to the burden of communicable diseases worldwide. Reportedly, much of infections and mortality from parasitic illnesses are restricted mainly in developing countries (157). Malnutrition or loss of appetite is a common characteristic of many infectious diseases including parasitic infections which result in reduced serum leptin levels (158). Since, leptin has been reported to induce pro-inflammatory cytokines & chemokines, neutrophil chemotaxis, NK cell cytotoxicity, and T cell functions, therefore its deficiency leads to an increase in susceptibility to infectious diseases (64, 65, 159, 160). Furthermore, leptin exerts central effects on hypothalamic-pituitary function and disruption of these effects have been implicated into severe parasitic diseases due to immune dysfunction in host (35–38).

Moreover, very little is known about the role of leptin in pathogenesis of parasitic infections. There is a need for studying the role of leptin in controlling parasitic infections since preponderance of such infections is associated with malnutrition which goes hand in hand in developing countries. As a first step, we have highlighted some of these studies to generate interest in initiating such studies (Table 1).

### Leishmaniasis

Leishmaniasis is a vector-borne protozoan disease caused by Leishmania parasites. Leishmaniasis is commonly prevalent in tropical and subtropical regions of the world with different immunopathology and varying degrees of morbidity and mortality. Among which, Visceral Leushmaniasis (VL) is the deadliest form of the diseases, marked by uncontrolled parasitemia in the spleen, liver, and bone marrow (161, 162).

VL is an endemic disease found mostly in economically poor societies, who invariably suffer from malnutrition. Malnutrition is characterized by the lower serum leptin level which in turn adversely alters the development of innate and adaptive immune responses during VL in both mice and children living in endemic areas (163–165). Hence, malnutrition and low serum leptin level are playing a critical role in *Leishmania* infection and its pathogenesis. Leptin improved cytokine production and phagocytosis of *Leishmania donovani* by murine and human macrophages by increasing the phagolysosome formation and oxidative killing of the parasite via intracellular reactive oxygen species (ROS) generation (36). Leptin in combination with miltefosine, the conventional antileishmanial drug, augments the protective immunity in mouse macrophage during *L. donovani* infection *in vitro* (130). Similarly, it enhances the host protective Th1 cytokine responses in THP-1, and human PMBCs derived macrophages by inducing Erk1/2 and Akt phosphorylation, which is usually dephosphorylated in *L. donovani* infection (36). It can also maintain the protective environment against *L. donovani* infection through the classical macrophage activation (36). Recently we demonstrated that leptin induces the innate immune response in bone marrow-derived antigen presenting dendritic cells, and causes heightened nitric oxide, proinflammatory cytokines (IFN-γ, IL-12, and IL1β) in the splenocytes stimulated with soluble Leishmania antigen. Besides this, leptin-induced IFN-γ production from both CD4+ and CD8+ T cells compared with untreated infected normal mice, indicates that leptin-induced heightened Th1 response (131). Alternatively, leptin deficient *ob/ob* mice had higher splenic and liver parasite burden compared with the normal infected mice. Nevertheless, leptin treatment of *ob/ob* mice failed to reduce the splenic parasite burden and host-protective cytokine response. Moreover, in contrast to DCs from a normal mouse, *ob/ob* mouse-derived DCs showed limitation in the initiation of innate immune response during *Leishmania* infection that could not be restored by leptin treatment suggesting that leptin signaling was differentially regulated in *ob/ob* mice compared with normal mice fed with healthy diet (131, 166). Interestingly, very recently we demonstrated that leptin also induces a protective CD8+ T-cell dependent immune response in malnutrition coupled with *L. donovani* infection through up-regulation of Granzyme A (GrzA) and down-regulation of cytotoxic T-lymphocyte-associated protein 4 (CTLA-4) and Programmed death-1 (PD-1) markers (132). The PD-1 ligand plays a significant role in CD8+ T cell exhaustion during the chronic infections of various infectious diseases including VL (167). It is worth mentioning here that in contrast to the above reports an increased expression or activity of leptin, has been reported in blood samples of dogs with canine leishmaniasis (CanL) and suggested possible use of leptin as a biomarker for CanL (168). In conclusion, leptin treatment may improve parasite clearance in malnourished VL condition through restoration of normal immune cell function via leptin signaling.

### Trypanosomiasis

Chagas disease is caused by a protozoan parasite *Trypanosoma cruzi*. The parasite is transmitted to humans and other hosts mainly by feces of infected blood-feeding triatomines, blood transfusion, or by ingestion of contaminated food (169). Chagas disease remains a serious health problem in Central and South America and a major cause of morbidity and mortality (135, 170). *T. cruzi* uses adipocytes as a reservoir for chronic infection and displays a pro-inflammatory phenotype by upregulating cytokines such as IL-1β, IFN-γ, TNF-α, and chemokines such as CCL2, CXCL10, and CCL5 along with innate immune receptors such as Toll-like receptor (TLR)-2 and 9 (171–173). Other pathways, such as ERK and PI3K pathways were also activated upon *T. cruzi* infection (174, 175). Additionally, Adipose tissue profoundly expressed Peroxisome proliferator-activated receptor (PPAR-γ) along with adiponectin, which exerts an anti-inflammatory effect. The levels of PPAR-γ were also decreased in *T. cruzi* infected cells, which leads to the reduced secretion of adiponectin and increased inflammatory reactions (176).

Since parasite infects many organs including adipose tissue which is a source to a variety of adipokines, including leptin and could have significant role in pathogenesis of Trypanosomiasis (133, 134). Mice infected with *T. cruzi* showed significant reduction in leptin levels, possibly due to adipocyte involvement in disease progression (133, 135). It was also reported that chemically induced diabetic mice and genetically susceptible *db/db* diabetic mice with defective leptin receptors had higher parasitemia and mortality after *T. cruzi* infection, which suggests that the dysregulation of host metabolism may be beneficial to parasitic survival in the host (136). Moreover, mice with defective leptin receptor are metabolically challenged and upon infection with *T. cruzi* suffer high mortality (37). In NSE-Rb *db/db* mice, a genetically modified *db/db* mouse, central leptin signaling is reconstituted only in the brain which is sufficient to correct the metabolic defects and when infected with *T. cruzi* showed reduced parasitemia, mortality rates, and tissue parasitism as compared to normal *db/db* mice. The plasma levels of several cytokines and chemokines were also significantly increased in infected *db/db* mice compared with NSE-Rb *db/db* mice (37). In summary, the normalization of the metabolic dysfunction in NSE-Rb *db/db* mice through the restoration of leptin receptor signaling in brain reconstitute the normal immune response against *T. cruzi* infection, but not peripheral restoration, highlighting that leptin may play a role as a central regulator for both metabolic function and immune response.

### Amoebiasis

Amoebiasis is the disease caused by an enteric protozoan parasite *Entamoeba histolytica*. Infection results from ingestion of the parasite cyst from feces-contaminated food or water (177). *E. histolytica* primarily lives in the intestinal mucosa and mainly restricted in colon infection causing devastating dysentery, colitis, and liver abscess by producing tissue damages (178, 179) while many deaths are associated with extraintestinal invasive disease (180, 181). Amoebiasis occurs when trophozoites disrupt the mucosal barrier and penetrate the underlying tissue and break down extracellular matrix, destroy cells, and phagocytose cellular debris (182). Studies on amoebic liver abscess (ALA) carried out in Indian subcontinent suggest that malnutrition is associated with ALA outcome (183, 184).

Moreover, malnutrition and serum leptin levels are directly proportional to the pathogenesis of amoebiasis, and low serum leptin plays a critical role in *E. histolytica*- associated diarrheal illness and extent of liver injury (137, 185). The mucosal immune response can be suppressed by a mutation in leptin receptor and defective STAT3 signaling pathways, resulting in susceptibility to intestinal abscess due to *E. histolytica* infections (38, 138). The mechanism of mucosal immune suppression depends on homozygous allelic mutation in leptin receptor Q223R (rs1137101) that ablates STAT3 signaling, results in decreased mucosal chemokine's LIF, CXCL9, CXCL10, CCL3, and CCL4 secretion (68, 69). A mutation or polymorphism in leptin receptor at 233 (from glutamine to arginine) is liable to enhance 4 times more susceptibility to *E. histolytica* infection in children irrespective of nutritional status (139). The leptin-deficient (*ob/ob*) and LepRb-deficient (*db/db*) mice were highly susceptible to infection with *E. histolytica*, whereas wild-type C57BL/6 mice were resistant. Moreover, mice either homozygous or heterozygous for the 223R allele of leptin receptor were significantly more prone to amoebic infection. Both types of mice ceca were shown to have profound epithelial denudation because of trophozoites invasion. Leptin signaling in the intestinal epithelium and downstream STAT3 and SHP2 (Src homology phosphatase 2) signaling was required for protection in the murine model of amoebic colitis (140). Leptin-mediated specific activation of STAT3 and ERK or Akt signaling pathways in gut mucosal epithelial cells offers more resistance against amoebiasis caused by *E. histolytica* infection (141, 142). In conclusion, leptin may control the amoebic infections by the activation of leptin signaling pathway in gut mucosal epithelial cells via up-regulation of various signaling pathway.

### Malaria

Malaria is caused by *Plasmodium* species, an intracellular parasite transmitted by the bite of an infected female Anopheles mosquito. It is endemic in most of the tropical countries such as sub-tropical regions of Asia, Africa, South and Central America (186). *Plasmodium falciparum* is the most virulent form of the human malaria parasites and responsible for 90% of malaria-related morbidity and mortality (187). During a mosquito bite, sporozoites are injected into host's skin, enter the bloodstream and reach to the liver. Parasites differentiate and replicate inside hepatocytes, and then released as merozoites into the bloodstream, which subsequently invades red blood cells (RBCs) (186).

Inflection of diet can affect the outcome of parasitic diseases either through the effects on parasite growth and development, or via the host immune response, or both. Leptin is a cytokine predominantly secreted by adipocytes that increases in proportion to total body fat mass, and upon exposure to pro-inflammatory cytokines, it inhibits both appetite and adiposity in malaria infection (39). Moreover, serum leptin levels were approximately five-fold higher in *Plasmodium berghei*-infected mice than in non-infected controls (188). Leptin-deficient mice infected with *P. berghei* ANKA were shown to be resistant to the development of cerebral malaria whereas the normal mice developed signs of cerebral malaria. Dietary restriction prevented severe experimental cerebral malaria (ECM) symptoms and death in mice through modulation of leptin levels and mechanistic target of rapamycin complex 1 (mTORC1) activity in T cells (143). Pharmacological inhibition of either leptin signaling with a mutant peptide, or downstream mTORC1 signaling with rapamycin, blocked ECM symptoms and reduced mortality (39). Furthermore, leptin exerts central effects on hypothalamic-pituitary function and these outcomes might affect the severity of malaria disease. Disturbance in hypothalamic-pituitary-adrenal axis during *P. falciparum* infection have been involved in the pathogenic mechanism of severe malaria (144). Importantly, the level of leptin in serum of malaria patients has been recently reported to be used as prognostic markers of treatment outcomes and pathogenesis of malaria patients (144). In conclusion, leptin could play an important role to control the immuno-compromised malarial infections by the activation of immune cells through leptin signaling pathway.

## Conclusion and summary

Many studies have been conducted in recent past to understand the role of leptin in immune modulation such as activation of phagocytosis, cytokine polarization and cell-mediated immunity in infectious diseases. Both obesity and malnutrition are pandemics associated with immune-deficiencies that lead to increased vulnerability to infectious disease. Interestingly, both obesity and malnutrition are related to aberrant leptin levels, obesity due to chronically elevated leptin levels, whereas malnutrition results in significantly diminished leptin levels. Emerging data from animal models and human indicates that immune dysfunction underlies the etiology of malnutrition and reduced immune-mediated protection from infections, which interplay between nutrition, leptin levels and immune responses (86, 189, 190). Malnutrition is characterized by immune suppression and increased risk of mortality from infectious diseases (191). The immune dysfunction is not only a consequence of inadequate diet but also contributes in various mechanisms, including the energy homeostasis, metabolism, the role of leptin and it signals to the hypothalamic-pituitary-adrenal axis and peripheral organs. Thus, it is likely that the CNS plays a critical role in malnutrition associated immune deficiency. Protein-energy malnutrition reduces leptin concentrations which impairs macrophage functions, ability to engulf pathogens and to produce proinflammatory cytokines (25, 27). Importantly, leptin has a crucial role in mediating innate and adaptive immune response which are significantly affected by nutritional status and play a vital role in the immune adaptation in both malnutrition and infection. Moreover, many infectious diseases directly or indirectly are linked to malnutrition which compromises the innate and adaptive immunity of host and increased susceptibility to infectious disease.

**Figure 2:**
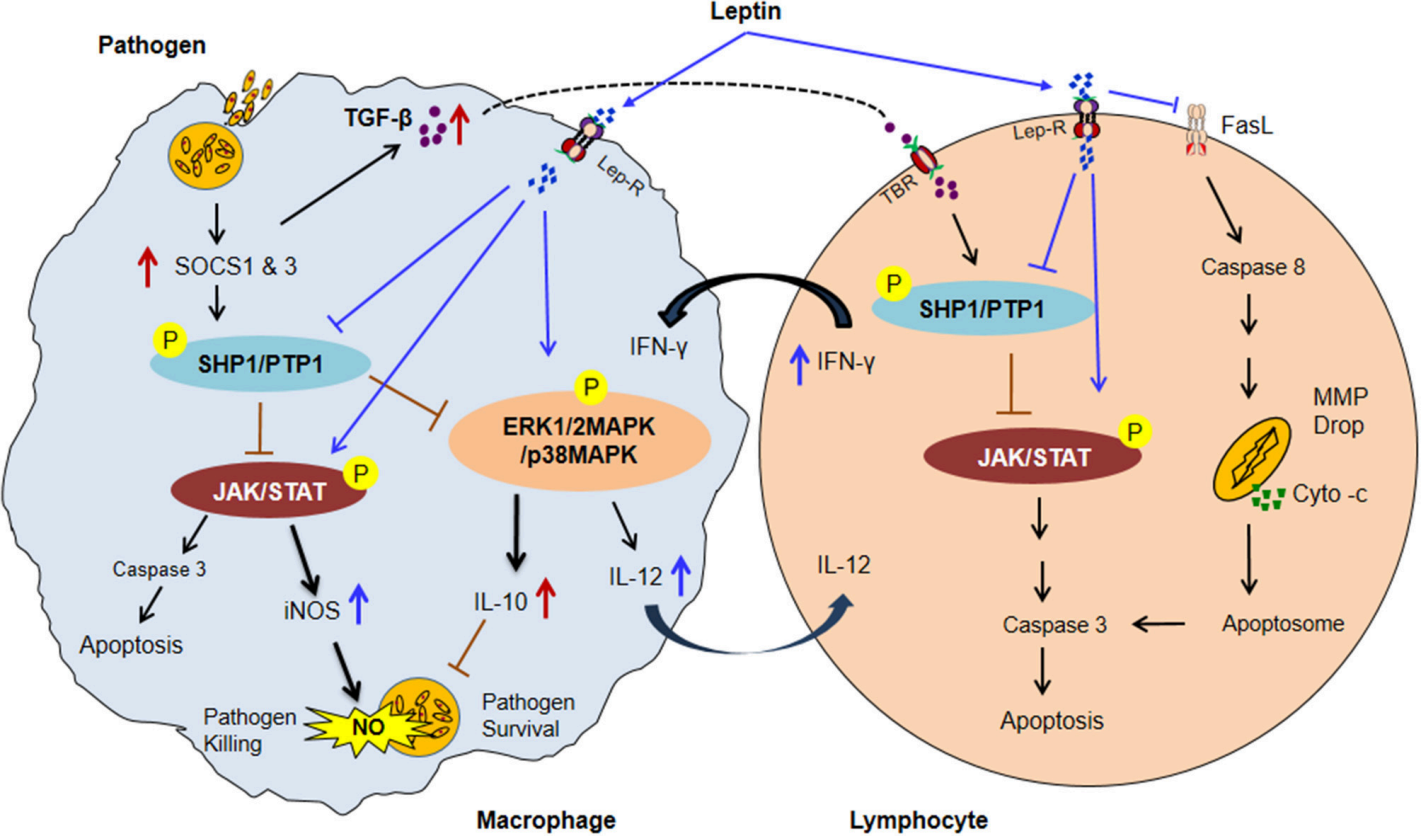
The possible model of Leptin and Immune response in malnutrition coupled infectious diseases. In malnutrition, low adipocyte mass causes a reduction of serum leptin level resultant impairment of normal macrophages and lymphocytes activities. Infected macrophages induce the SOCS1 & 3 proteins expression subsequently upregulates ROS scavenging enzyme Thioredoxin which leads to activates SHP1/PTPase molecules. SHP1/PTP1 negatively regulates the JAK/STAT, and MAP-Kinase pathways thus inhibiting IFN–inducible macrophage functions (increased IL-10 and TGF-β level and decreased the IL-12 cytokines in infected macrophage). IL-10 suppresses the NO activity and improves the parasite survival. TGF-β activates SHP1/PTPase activity in lymphocytes through TGF-β receptor (TBR) which leads to lymphocytes apoptosis. In contrast, Leptin treatment inactivated SHP1/PTPase directed pathways and reversed the macrophage activities by up-regulating the pro-inflammatory cytokines (IFNγ, TNF-α, and IL-12) secretion and NO expression. IL-12 cytokine released from activated macrophage upon leptin treatment inhibits the SHP1/PTPase dependent T lymphocytes apoptosis by activation of JAK/STAT pathway. Moreover, Leptin directly inhibits the FasL-dependent T lymphocytes apoptosis by the inhibition of the caspase 8 activity. Caspase-8 then promotes mitochondrial outer membrane permeabilization (MOMP) by diminishing the inhibitory effect of various antiapoptotic and proapoptotic molecules. MOMP results in cytochrome-c release from the mitochondria, enabling activation of a supramolecular complex, the apoptosome that activates caspase-3 to undertake apoptotic cell death (Suppressors of cytokine signaling: SOCS1 & 3; Protein tyrosine phosphatases: SHP1/PTP1, Mitochondrial membrane potential drop: MMP drop, and P: phosphorylation) (192–206).

More importantly the mechanism of leptin signaling in various infectious diseases is depends on SOCS3 expression as describes in NF-κB dependent pathway. The SOCS3 expression attenuates the macrophage's response to IFN-γ at both proximal level activation and downstream expression. Hence, taken together above-mentioned observations indicate that the potential role of leptin signaling in various pathogens has been summarized in a schematic diagram (Figure 2) (192–205). Therefore, understanding the link between nutrition, leptin, and immune dysfunction in murine and human infectious diseases will inform targeted interventions for a vulnerable population with undernutrition, which is a crucial need for new approaches to reduce global mortality from infectious diseases. Present review provides a rationale for future studies to explore role of leptin as therapeutics to host immune dysfunction in infectious diseases during malnutrition.

## Author contributions

RM conceived and conducted literature reviews, figure, and table construction and contributed to the writing of the manuscript. PB, RD, and HN critically edited and reviewed the complete manuscript, tables and figures. All authors approved the final manuscript for submission.

### Conflict of interest statement

The authors declare that the research was conducted in the absence of any commercial or financial relationships that could be construed as a potential conflict of interest.
